# Treatment outcome of new pulmonary tuberculosis in Guangzhou, China 1993–2002: a register-based cohort study

**DOI:** 10.1186/1471-2458-7-344

**Published:** 2007-11-29

**Authors:** Qing-Song Bao, Yu-Hua Du, Ci-Yong Lu

**Affiliations:** 1 Department of Medical Statistics and Epidemiology, School of Public Health, Sun Yat-Sen University, Zhongshan Road II 74, Guangzhou, Guangdong Province, The People's Republic of China; 2 The Chest Hospital, Guangzhou, Heng-zhi-gang Road 62, Guangzhou, Guangdong Province, The People's Republic of China

## Abstract

**Background:**

Completion of treatment for tuberculosis (TB) is of utmost priority for TB control programs. The aims of this study were to evaluate the treatment outcome of TB cases registered in Guangzhou during the period 1993–2002, and to identify factors associated with treatment success.

**Methods:**

Two (of eight) districts in Guangzhou were selected randomly as objects of study and their surveillance database was analyzed to assess the treatment outcome and identify factors associated with treatment success for TB cases registered in Guangzhou. Six treatment outcome criteria were assessed based on guidelines set by the World Health Organization (WHO). Logistic regression was used to estimate risk factors for treatment outcome.

**Results:**

A total of 6743 pulmonary tuberculosis cases (4903 males, 1840 females) were included in this study. The treatment success rate (including cured and complete treatment) was 88% (95%CI 87%–89%). One hundred and eight-six (2.8%) patients died and 401 (5.9%) patients defaulted treatment. In multivariate analysis, treatment success was found to be associated with young age, lack of cavitation and compliance with treatment.

**Conclusion:**

The total treatment success rate in the current study was similar to the WHO target for all smear positive cases, while the failure rate and the default rate in 2002 were slightly higher. Good care of elderly patients, early diagnosis of cavitation and compliance with treatment could improve the success rate of TB treatment.

## Background

Worldwide, TB is second only to HIV/AIDS as a cause of death from infectious disease. There were 8.9 million new cases of TB in 2004 (140/100 000 population), of which 3.9 million (62/100 000) were smear-positive and 741 000 were in adults infected with human immunodeficiency virus (HIV). An estimated 1.7 million people (27/100 000) died from TB in 2004, including those co-infected with HIV (248 000) [1]. It has been estimated that TB ranks seventh among all illnesses as a cause of disability adjusted life years (DALYs) lost, an estimate of disease morbidity, and it is projected that ranking is unlikely to change through the early part of the twenty-first century [2].

The scale of the global TB epidemic demands urgent and effective action. It is very important in tuberculosis (TB) control to detect the disease as early as possible and to ensure that those diagnosed complete their treatment and get cured. The World Health Organization (WHO) target for treatment success is 85% of all detected smear-positive cases [3]. Even where free medication is available, many patients are not successfully treated [4,5]. Main reasons for non-success are death (while on treatment or before start of treatment) and loss to follow-up. Incomplete treatment may result in prolonged excretion of bacteria that may also acquire drug resistance, cause transmission of disease and lead to increased morbidity and mortality [6].

Monitoring the outcome of treatment is essential in order to evaluate the effectiveness of the intervention. Recommendations on how to evaluate treatment outcomes using standardized categories have been issued by the World Health Organization (WHO) in conjunction with the European Region of the International Union Against Tuberculosis and Lung Disease (IUATLD). WHO and IUATLD use an agreed set of six possible and mutually exclusive categories of treatment outcome in high-incidence countries. These categories are cured, treatment completed, failure, death, treatment interrupted, and transfer out [7,8]. Ideally, treatment outcomes in all patients should be routinely monitored by the epidemiological surveillance system. This would make it possible to recognize and amend system failures before the incidence and proportion of resistant isolates rise.

In 1991, the Government of China introduced a TB control project using the WHO-recommended, five-point strategy called Directly Observed Treatment Short-course (DOTS). The project, entitled the Infectious and Endemic Disease Control (IEDC) project, was assisted by a World Bank loan and was implemented in 12 provinces with a population of 573 million, roughly one-half of China's population in 1991. Following the implementation of DOTS, treatment outcomes were excellent and improved over time. There were about 4.1–4.9 million active tuberculosis patients (367/100 000 population) in 2000, of which 1.33–1.68 million (122/100 000 population) were smear-positive. Overall, the cure rate was 95% and 90% for new and previously treated (relapse and other re-treatment) cases, respectively. From the first to the sixth year of DOTS implementation, the cure rate for both new and previously treated cases improved, while the treatment failure rate and death rate both decreased. Roughly two-thirds of the eventual improvement in treatment outcomes took place between the first and second year of DOTS implementation. For example, the percentage of treatment failure among new cases declined from 2.8% to 0.5% over the first six to eight years of DOTS implementation, but this percentage declined from 2.8% to 1.2% during the first year alone[9].

As the third biggest city in China, Guangzhou has about 7.1 million inhabitants. The incidence of TB cases has increased from 88.8/100000 population in 1995 to 91.1/100000 population in 2003. As a part of the World Bank supported project in China, Guangzhou adopted DOTS since 1993. The aims of this study were to evaluate the treatment outcome for TB cases registered in Guangzhou during the period 1993–2002, and to identify factors associated with treatment success outcome.

## Methods

### Study design

This was a register-based cohort study. In China, there is compulsory nominative notification of all TB cases directly to the Ministry of Health of The People's Republic of China. Two of eight districts in Guangzhou were selected randomly as study objects and their surveillance database was analyzed to estimate the treatment outcome and factors associated with treatment success for TB cases registered in Guangzhou during the period 1993–2002.

### Data source

By law, TB is a reportable disease in Guangzhou. Once a TB case is diagnosed, a standard notification form would be completed by the treating physician and then entered into an electronic database. In our study, only pulmonary TB cases, but not extra-pulmonary TB cases were selected for analysis. Patients were admitted at the local district level and treated at TB treatment facilities or general hospitals. Data on prisoners and military personnel is not included in this database.

### Treatment regimens

All the patients diagnosed with tuberculosis were given treatment by the doctors at the local district hospitals or TB treatment facilities. The DOT and anti-tuberculosis regimens used in our study was in accordance with the China Tuberculosis Control Collaboration (CTCC) strategy [10]. Free drugs were prescribed thrice weekly. All patients had a tuberculosis treatment card. This card was sent to the doctor who supervised every dose of the regimen. Some work was done by the doctors, including pill counts, urine analysis and home visits, to ensure DOT completion.

### Definitions

The treatment outcome was defined according to the WHO and IUATLD guidelines, with some modifications [11,12]. These definitions were: Cure (person who became sputum-smear – negative in the last month of treatment and on at least one previous occasion), treatment failure (person who continued to have sputum-smear – positive status at >5 months during treatment), treatment completed (person with PTB+ who completed treatment but whose condition was not consistent with the criteria for either cure or failure), died (person who died of any cause during treatment, A patient who died with tuberculosis but never started treatment were included in the denominator), defaulter (person who interrupted the treatment regimen for >2 consecutive months), transfer out (person who moved to another health-care facility and was entered in a new diagnostic category, i.e., transfer in), treatment success (a patient who was cured or who completed treatment).

Death during the period 1993–2002 was determined using a combined review of the medical records and telephone follow-up. Results were further confirmed using the death certificate registry of the Department of Health.

Compliance with treatment based on the assessment of the treating physician was also noted. In this study, the researchers defined compliance by how many weeks the patients completed therapy, not the number of doses of medication. A trained graduate nursing research assistant collected data on compliance by pill counts, urine analysis and home visits. The patient was regarded as non-compliant when there was a record that he or she interrupted treatment for more than 2 weeks. It was also noted if a patient was advised to stop taking medicine because of adverse side effects. Patients were considered non-compliant if they did not come to the clinic and did not respond to a phone call and/or a letter from the public health nurse.

### Data analysis

Data were double entered into an Epidata (version 3.0) database and checked for errors by trained workers. Data analysis was performed using the Statistical Package for the Social Sciences version 13.0. Means and frequencies (%) were used to describe patients' characteristics. Odds ratios (OR) and their 95% confidence intervals (CI), were estimated using logistic regression, with TB treatment result as an outcome. The relation coefficient was used to assess the association between the explanatory variables and the treatment result, and to test for interaction and trend. Logistic regression analysis was used to study associations between the study-defined treatment outcome variables and sociodemographic and behavioral risk factors. First, univariable analyses were performed to examine the effect of each variable on the risk of TB. Then final multivariate logistic models were constructed, including variables that were significant at the two-sided alpha = 0.05 level in univariate analyses. A P value less than 0.05 was considered statistically significant.

## Results

During the period considered, a total of 6915 new tuberculosis cases were registered in the database; of these, 6743(97.5%) cases, which have complete outcome data available, were selected for our study. These new tuberculosis patients were notified with positive results, i.e. either positive sputum smear patients (2230, 33.1%) or culture positive patients (4513, 66.9%) (Table 1). Of all the cases, 5887(87.3%) patients were detected through passive case finding (due to their symptoms). A total of 803(11.9%) patients were identified through regular health check, 9(0.1%) patients were detected through follow ups of close contacts of identified infectious cases and another 9 patients were detected through the TB screening program. The rest (35 patients) had no detailed information about identified ways in the database.

**Table 1:** Treatment outcome of new tuberculosis patients by registered year, Guangzhou, 1993–2002

Registered year	Cured	Completed treatment	Died	Failure	Defaulted treatment	Transferred Out	Total
	No. (%)	No. (%)	No. (%)	No. (%)	No. (%)	No. (%)	
1993	355(52.1)	161(23.6)	22(3.2)	29(4.3)	111(16.3)	3(0.4)	681
1994	258(37.7)	289(42.3)	12(1.8)	28(4.1)	88(12.9)	9(1.3)	684
1995	228(32.9)	354(51.2)	11(1.6)	29(4.2)	69(10.0)	1(0.1)	692
1996	286(40.5)	374(52.9)	24(3.4)	10(1.4)	10(1.4)	3(0.4)	707
1997	312(41.3)	388(51.4)	25(3.3)	14(1.9)	13(1.7)	3(0.4)	755
1998	313(46.4)	321(47.6)	26(3.9)	5(0.7)	10(1.5)	0(0.0)	675
1999	251(40.8)	317(51.5)	21(3.4)	8(1.3)	16(2.6)	2(0.3)	615
2000	248(39.7)	331(53.0)	9(1.4)	13(2.1)	20(3.2)	3(0.5)	624
2001	270(41.7)	313(48.4)	19(2.9)	26(4.0)	19(2.9)	0(0.0)	647
2002	242(36.5)	326(49.2)	17(2.6)	27(4.1)	45(6.8)	6(0.9)	663
Total	2763(41.0)	3174(47.1)	186(2.8)	189(2.8)	401(5.9)	30(0.4)	6743

In this study, 2763(41%) patients were cured and 3174(47%) patients completed treatment, which gave a total treatment success rate of 88% (95%CI 87%–89%). The average duration of treatment was 6.5 months (Range: 6.0–20.2 months). Furthermore, the treatment success rate among smear positive patients (2230) were 86%(95%CI 85%–87%), Among all the 5937 patients who had treatment success outcome, 5774(97.3%) patents had only pulmonary tuberculosis, 163(2.6%) patients had both pulmonary tuberculosis and extra-pulmonary tuberculosis, and one patient had tuberculosis in more than two organs. A total of 5592 patients knew the accurate time when their symptoms started. The median of symptomatic interval prior to diagnosis and treatment was 6.48 weeks, which decreased from 9.68 weeks in 1993 to 5.52 weeks in 2002.

Altogether 186(2.8%) patients died, with 157(2.4%) who died while on treatment and 29(0.4%) who died from TB before treatment start. The median age at death from TB in the study was 71 years and the average duration of treatment was 75 days. For 106(57.0%) patients, TB was the primary cause of death. For the rest 80(43.0%) patients, TB was a contributing factor to their death.

There were 401 (5.9%) patients who defaulted treatment and the average duration of treatment was 16.4 weeks. Among these cases, 280 (69.9%) patients defaulted during the first 12 weeks of treatment, 268(66.8%) were reported in 1993–1995. Unfortunately, there was no systematic information for the reason they defaulted treatment.

During 1993–1995, the success rate gradually increased from 75.7% to 84.1%, and it then reached and stayed at 90% for the following six years. It is noted that the completion rate became higher (P < 0.001) during 1995–2002. Both the failure rate and the default rate dropped during 1993–1995. However, they went up again in 2002, with 27(4.1%) patents failed and 45(6.8%) patients defaulted treatment. In addition, 6(0.9%) patients were transferred out in 2002.

Of the 6743 cases, 4903(72.7%) patients were male and 1840(27.3%), female (Table 2). The treatment success rates for females and for males were 91% and 87% respectively. Un-successful outcomes were more common (P < 0.001) among males than among females: the death, failure and default rate among males were 3.2%, 3.2% and 6.4% respectively. However, gender turned out to be non-significant for treatment success outcome after multivariate logistic regression analysis (Table 3).

**Table 2:** Number of new tuberculosis patients by patient characteristics and treatment outcome, Guangzhou, 1993–2002

	Cured	Completed treatment	Died	Failure	Defaulted treatment	Transferred Out	Total
	No. (%)	No. (%)	No. (%)	No. (%)	No. (%)	No. (%)	
Sex							
Male	2037(41.5)	2127(45.4)	153(3.1)	153(3.1)	309(6.3)	24(0.5)	4903
Female	726(39.5)	947(51.5)	33(1.8)	36(2.0)	92(5.0)	6(0.3)	1840
Age group(yrs)							
0–14	12(15.0)	63(78.8)	1(1.3)	0(0.0)	4(5.0)	0(0.0)	80
15–39	1325(45.1)	1370(46.6)	5(0.2)	71(2.4)	154(5.2)	13(0.4)	2938
40–64	936(41.6)	1064(47.3)	49(2.2)	64(2.8)	133(5.9)	4(0.2)	2250
65+	490(33.2)	677(45.9)	131(8.9)	54(3.7)	110(7.5)	13(0.9)	1475
Cavitations							
No	1684(36.4)	2452(53.0)	107(2.3)	81(1.8)	279(6.0)	23(0.5)	4626
Yes	1079(51.0)	722(34.1)	79(3.7)	108(5.1)	122(5.8)	7(0.3)	2117
Compliance with treatment							
No	13(2.1)	23(3.8)	94(15.5)	86(14.2)	368(60.6)	23(3.8)	607
Yes	2750(44.8)	3151(51.4)	92(1.5)	103(1.7)	33(0.5)	7(0.1)	6136

**Table 3:** Odds ratio (OR) for treatment success vs. non-success among new tuberculosis patients, Guangzhou, 1993–2002

	Treatment Success No. (%)		Univariate analysis			Multivariate analysis*		
			
	Yes	No	OR	95%CI	P	OR	95%CI	P
Sex								
Men	4264(87)	639(13)	1.00			1.00		
Women	1673(91)	167(9)	1.50	1.26–1.80	<0.001	NS^a^	NS^a^	
Age group(yrs)								
0–14	75(94)	5(6)	3.96	1.58–9.87	0.003	3.52	0.80–15.43	0.095
15–39	2695(92)	243(8)	2.92	2.44–3.51	<0.001	3.36	2.47–4.56	<0.001
40–64	2000(89)	250(11)	2.11	1.76–2.53	<0.001	2.21	1.64–2.98	<0.001
65+	1167(79)	308(21)	1.00			1.00		
Cavitations								
No	4136(89)	490(11)	1.00			1.00		
Yes	1801(85)	316(15)	0.67	0.58–0.78	<0.001	0.51	0.39–0.65	<0.001
Compliance with treatment								
No	36(6)	571(94)	1.00			1.00		
Yes	5901(96)	235(4)	398.3	277.5–571.5	<0.001	462.1	317.1–673.5	<0.001

*In the multivariate analysis, all variables in the univariate analysis were considered

^a^NS not significant

The median age of patients in our study was 42 years (range 1–96 years) overall. The proportion of treatment success differed by age group: completed treatment outcomes were more frequent below age 14; both cured outcomes (490 patients, 33.2%) and completed treatment outcomes (677 patients, 45.9%) were lower above age 65.

In our study, 2117 patients had cavitations. Among these patients, 1079(51.0%) were cured; 722(34.1%) completed treatment; 79(3.7%) patients died; 108(5.1%) patients failed treatment, 122(5.8%) patients defaulted and 7(0.3%) patients were transferred out.

Compliance with treatment based on the assessment of the treating physician was also noted. There were 6136 patients who were compliance in our study. Among these patients, 2750(44.8%) patients were cured; 3151(51.4%) patients completed treatment; 93(1.5) patients died; 103(1.7%) patients failed treatment; 33(0.5%) patients defaulted and 7(0.1%) patients were transferred out.

The odds ratio from logistic regression is presented in Table 3. When adjusted for all other predictors, gender was no longer significant. In the 15–39 age group, treatment success outcome was 3.08 times (95% CI 2.26–4.19) more than in the reference age group above 65. Among the patients with cavitations, treatment success outcome was 0.69 times (95% CI 0.533–0.91) less than among the patients without cavitations. Patients who complied with treatment had high odds of success both in the univariate analysis (OR = 398.3, 95%CI 277.5–571.5) and multivariate analysis (OR = 462.1, 95%CI 317.1–673.5).

## Discussion

The tuberculosis control project DOTS, funded by a loan from the World Bank, has been carried out by Guangdong province, China since 1993. Here our study was designed to assess the results of treatment for TB within the nine years after the implementation of this system. Overall, the treatment success rates among smear positives were 86% (95%CI 85%–87%). This number meets the WHO targeted success rate of 85% for all smear positive cases. However, problems still exist, for example, the low treatment success rate and the high default rate in 2002. Some studies have suggested that HIV is the strongest predictor of progression from latent TB infection to active disease [13-16]. Because HIV test is not required for TB cases in Guangzhou and data was obtained from TB surveillance database, we have no detail information about HIV prevalence in this study. However, according to routine monitoring data, the number of HIV-infected patients of Guangzhou had increased rapidly since 1995, and reached 576 in 2002 from 22 in 1992 [17,18]. Although how much it contributed to higher unsuccessful rates in 2002 could not be determined, the emergencies of more interaction of TB with human immunodeficiency virus (HIV) infection might be one of the reasons.

In our study, 803(11.9%) of TB patients were identified through health check and 9(0.1%) through investigation of contacts of cases. These findings indicate that screening programs to detect new TB cases were limited in Guangzhou city. However, it is difficult to expand the current scale of screening in such a large city due to the large number of fluid population which increased to 3.31 million in 2000. Management of fluid population is incomplete since many of them have no stable work and address. More extensive screening of high risk populations, e.g. fluid population, contacts of cases etc, needs to be implemented.

The death rate in our study was 2.8% (95% CI 2.4%–3.2%), is lower than those reported previously by several similar studies, including 24%, 14%, 9% and 6% in Baltimore City, USA [9], Vaud County, Switzerland [5], Norway [19] and Hamburg, Germany [20] respectively. However, common in those studies, most patients who died from TB were old (median age was 71 years). Our logistic regression models confirmed that advanced age is one of the significant risk factors for non-successful treatment, since advanced age itself contributes to higher mortality, partly through co-existing illness. One study suggested that treatment of latent TB in such high risk elderly patients should be a high priority, although advanced age is a relative contraindication [21]. Other studies recommended starting anti-TB treatment on suspicion whilst awaiting results of diagnostic tests in elderly patients, provided there is no other obvious cause of their illness [22,23]. Our study also found 29 patients who died before the start of treatment. These high risk patients should be paid more attention.

Our study showed a total default rate of 5.9% for new pulmonary TB. Higher default rates have been described in other studies such as Vaud County, Switzerland (16%) [5], Hamburg, Germany (10%) [20] and Sweden (7%) [4]. Although the default rate in this study is lower, defaulted patients constitute a major problem in pulmonary TB treatment management.

According to health regulations in China, hospitals are supposed to refer TB suspects and patients to the TB dispensary after they are identified. But the patients may prefer to be treated by the hospitals, which are frequently considered to have more clinical expertise than the TB dispensaries. Therefore, in spite of the regulation to refer patients and providers were paid a TB case-referral fee by the project, some patients change their treatment institution after referred to the TB dispensary. Unfortunately, we have no data about how many of patients did that. In addition, there is an economic incentive for the hospitals to keep and treat TB suspects and patients, because they could charge patients for their TB-related diagnosis and treatment. Furthermore, frequent change of address and social stigma related to TB might also be the reasons for default.

Our study found that cavitation is negatively associated with treatment success outcome (OR = 0.51, 95% CI 0.39–0.65). Of 2117 patients with cavitations, 108(5.1%) patients failed in tuberculosis treatment. According to several reports, progressive cavitations generally develop after several months with or without the development of an intracavitary mycetoma [24,25]. For these patients with cavitations, we could not determine the accurate time that pathological changes occurred because 94.1% (1992/2117) of them were detected through their symptoms. At the time they began tuberculosis treatment, the cavity had already formed and their tuberculosis was very severe. Therefore, non-successful outcomes were more common among those patients with cavitations in our study.

The logistic regression test also demonstrated that compliance could significantly influence treatment outcome. The risk of treatment success outcome was higher (OR = 462.1, 95% CI 317.1–673.5) among patients who complied with treatment than those who did not. Compliance played an important role in the outcome of tuberculosis treatment. To improve patient compliance, some measures can be taken. First of all, public education, such as publications, kits, workshops, reports, videos on TB and the process of the study itself are necessary to improve public awareness and knowledge of the prevalence and treatment of tuberculosis. Second, the dispenser is a key person in the health system to ensure adherence by communicating with patients about the use of medication. A qualitative study from South Africa reported that training health care providers in communication skills brought about a significant shift towards a more patient-centered approach, resulting in the mutual satisfaction of patients and health providers [26]. So we recommend that training to enhance communication skills should be introduce at TB clinics and hospitals. Third, lack of free treatment is often related to non-compliance [27,28]. Therefore, government should provide adequate finance support to carry out DOTS and extend its coverage. Furthermore, TB patients and their relatives should receive regular counseling from specially trained health workers. Health professionals should anticipate critical moments in treatment adherence and assist the patient and his/her family to bridge them.

This study was hindered by some unavoidable limitations. The Guangdong government provided the surveillance database and outcome data of the registered TB patients. We have no detail information of every patient in this study. However, this is beyond our control. Also, because of the difficulty of contacting the defaulters, our study could not address some of the factors leading to default. Further studies to explore the various factors involved in irregular TB treatment are needed. Nevertheless, our findings are applicable to the current situation of TB control in Guangdong province, and may draw attention of physicians dealing with tuberculosis to the problems that could be encountered in the management and control of TB.

## Conclusion

The total treatment success rate in the current study was similar to the WHO target of success rate for all smear positive cases, but the failure rate and default rate were slightly high in 2002. Good care of elderly patients, early diagnosis in patients with cavitations and compliance with treatment could improve the success rate.

## Competing interests

The author(s) declare that they have no competing interests.

## Authors' contributions

C-YL conceived of the study, and participated in its design and helped to draft the manuscript. Q-SB participated in the study design and performed the statistical analysis and drafted the manuscript. Y-HD collected the data and helped to draft the manuscript.

## Pre-publication history

The pre-publication history for this paper can be accessed here:


